# An American Text-Book of Surgery

**Published:** 1893-01

**Authors:** 


AN AMERICAN TEXT-BOOK OF SURGERY FOR PRACTITIONERS AND STUDENTS. By Charles H. Burnett, M. D., Phineas S. Conner, M. D., Frederic S. Dennis, M. D., William W. Keen, M. D., Charles B. Nancrede, M. D., Roswell Park, M. D., Lewis S. Pilcher, M. D., Nicholas Senn, M. D., Francis J. Shepherd, M. D., Lewis A. Stimson, M. D., William Thomson, M. D., J. Collins Warren, M. D., and J. William White, M. D. Edited by William W. Keen, M. D., and J. William White, M. D. One volume, twelve hundred pages, profusely illustrated. Philadelphia: W. B. Saunders, 913 Walnut Street. 1892. Sold by subscription only. Price, cloth, $7.00 net; sheep, $8.00 net; half Russia, $9.00 net.

This is the latest and most magnificent book upon surgery it has been the fortune of this editor to see. It covers the whole domain of surgery and is finely illustrated, not only with wood cuts, but with plates which are copies of photographs from nature.

That the reader may get a general idea of the scope of the work, we quote from the table of contents. It is divided into four books. Book I relates to general surgery; Book II, special surgery; Book III, regional surgery; and Book IV, operative surgery. In these four books are comprised forty-seven chapters. The first chapter opens with an elaborate and highly-interesting account of the germ theory, under the title "Surgical Bacteriology." In this chapter the attentive student will gain a complete survey of the present state of knowledge concerning bacteria and bacilli. The second chapter gives the whole theory of inflammation, while chapter III supplements it with a minute account of the process of repair. The traumatic fevers, suppuration, and abscess, ulceration, and fistula, gangrene, septicemia, erysipelas, tuberculosis, and syphilis occupy the succeeding chapters of this first book. Book II, special surgery; gives the surgery of the vascular system, osseous system, fractures, orthopædic surgery, etc. Book III, regional surgery; gives a separate chapter each to surgery of the head, spine, respiratory organs, neck, digestive tract, abdomen, genito-urinary tract, breast, eye, and ear. Book IV, operative surgery; gives, in successive chapters, anæsthesia, plastic surgery, ligation of arteries, operations on bones and joints, amputations, and minor surgery.

It will thus be seen that there is within the one volume that comprises this noble work a complete encyclopædia of the present state of knowledge in the practice of surgery. No physician, no matter whether he practices surgery or not, should continue in his profession without making himself familiar with the first book of this treatise.

We are in receipt of a letter from the publisher, in which he says:

"The *American Text-Book of Surgery*, edited by Professors Keen and White, of Philadelphia, which has only been issued a few months, is already a phenomenal success. It has been adopted as a 'text-book' by forty-nine of our leading medical colleges and universities. Nearly five thousand copies have been placed in physicians' libraries, and every indication points to a sale of at least as many copies more in the next six months.

"Dr. Nicholas Senn, of Chicago, is now preparing a 'Syllabus of Lectures on the Practice of Surgery' arranged in conformity with the *American Text-Book of Surgery*, which will be a valuable aid to all who have this great book."

